# Supplementary material for: Dependency of B-Cell Acute Lymphoblastic Leukemia and Multiple Myeloma Cell Lines on MEN1 Extends beyond MEN1–KMT2A Interaction
Source: Int J Mol Sci. 2023 Nov 17;24(22):16472. doi: 10.3390/ijms242216472 (PMC10670986; doi:10.3390/ijms242216472)
Supplement: Supplementary file 1 [file ijms-24-16472-s001.zip › ijms-2700658-supplementary.pdf]

**Supplementary Material.**

**Supplementary Figure S1**

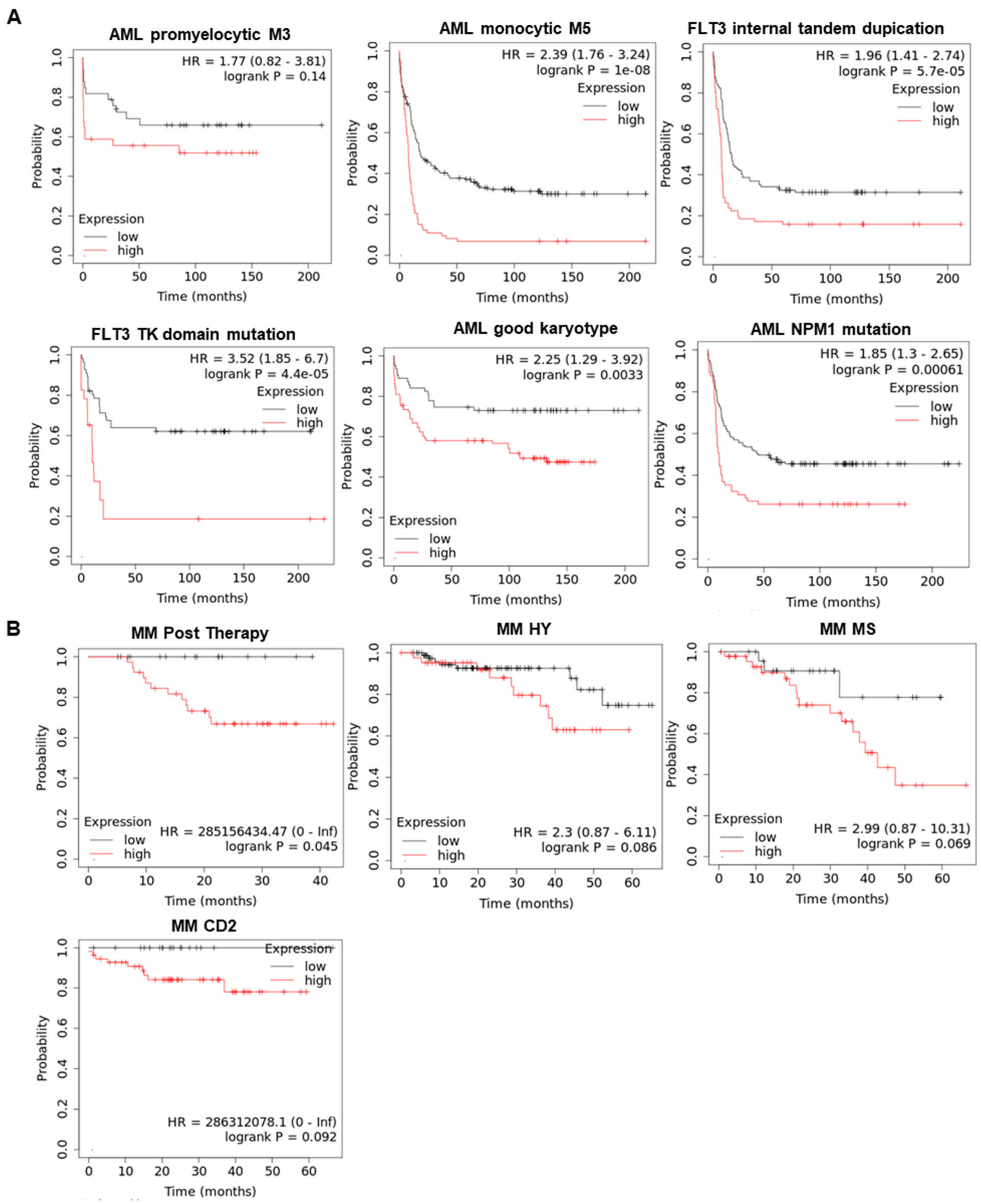


**Supplementary Figure S1.** Kaplan-Meier analysis of the association of overall survival with MEN1 expression in AML and MM sub-groups and mutational variants. AML and MM all subtypes were mined and analyzed by Kaplan-Meier Plotter https://kmplot.com/analysis/index.php?p=service), MEN1 probe set 202645_s_at. Cutoff: median. HR – hazard ratio. (**A**) Association of the worse survival with high MEN1 expression in

subtypes and mutational variants of AML. AML "good" karyotype (<3 chromosomal aberrations); data sets GSE1159, GSE6891, low n=63, high=94; P=0.0033. AML promyelocytic M3. Data sets: GSE1159, GSE37642, GSE6891; low n=33, high n=34; P=0.14. AML monocytic M5. Data sets: GSE1159, GSE37642, GSE6891; GSE12417; low n=182, high n=73; P=$1x10^{-8}$. AML NPM1 mutated. Data set: GSE1159, GSE6891; low n=151, high n=65; P=0.00068. AML FLT3 internal tandem duplication mutation, Data sets GSE1159, GSE6891; low n=114, high n=76; P=$5.7x10^{-5}$. AML tyrosine kinase domain mutation, Data sets GSE1159, GSE6891; low n=56, high n=23; P=$4.4x10^{-5}$. AML NPM1 mutation, Data sets GSE1159, GSE6891; low n=151, high n=65; P=0.00061. (**B**) Association of the worse survival with high MEN1 expression depending on treatment protocol (TT6, probe set GSE57317) and molecular subtypes (MS, HY, and CD2, probe sets GSE4204, GSE57317) in MM. MM TT6, low n=16, high n=39; P=0.045. MM MS, low n=25, high n=45; P=0.069. MM HY, low n=83, high n=44; P=0.086. MM CD2, low n=83, high n=44; P=0.086.

**Supplementary Methods**

**Cell Lines and Treatment**

B-ALL cell lines NALM-6, RS4;11, SUP-B15, were cultured in complete RPMI1640 medium containing 20% of FCS (Gibco, Waltham, MA, USA). In addition to FCS complete medium was complemented with 2 mM L-glutamine, 100 U/mL penicillin, 100 μg/mL streptomycin, and 50 μM monothioglycerol for the MM cell lines. KOPN-8 in complete medium containing 10% FCS. MM cell lines U266; OPM2, and MM1.s were cultured in RPMI 1640 medium containing 10% FCS, KMS-12-BM 20% FCS, and L363 15% FCS. LP1 cells were cultured in IMDM medium (PAN-Biotech, Aidenbach, Germany) containing 20% FCS. The HEK293T cells were cultured in complete DMEM medium (Gibco) containing 10 % FCS. All cells were cultured at 37°C and 5 % $CO_2$. Cell lines were authenticated by short tandem repeat (STR) DNA typing using GenomeLab™ GeXP Genetic Analysis System (Sciex, Darmstadt, Germany) and GenomeLabHuman STR primer set (Beckman Coulter, Brea, CA, USA) or experiments were performed right after receipt from DSMZ. STR profiles were analyzed using the "Online STR Analysis" tool provided by DSMZ (comprehensive DSMZ database of STR cell line profiles, www.dsmz.de) and the ExPASy bioinformatics resource portal database "Cellosaurus" (https://web.expasy.org/cellosaurus/; RRID: SCR_013869). Mycoplasma contamination was controlled using the Mycoplasma Detection Kit-Quick-Test (Biotool.com, Absource Diagnostics, Munich, Germany). Cells were counted and their viability was analyzed with help of the trypan blue dye exclusion method using the Vi-CELL XR cell viability analyzer (Beckman Coulter),

## ShRNA-Mediated Knockdown of MEN1

The shRNA target sequences for MEN1 were mined from Broad Institute RNAi consortium shRNA library (https://www.broadinstitute.org/rnai-consortium/rnai-consortium-shrna-library). As a negative control we used a sequence that does not have targets in the human genome {Sarbassov, 2005 #184}. Corresponding oligonucleotides were assembled and cloned into the BsbI and EcoRI cutting sites of pRSI12-U6-sh-UbiC-TagRFP-2A-Puro (Cellecta, https://www.cellecta.com/) using the Rapid DNA Ligation Kit (Roche, Basel Switzerland). ShRNA target sequence as well as amplification primers are listed in the Supplemental Table S1.

**Supplemental Table S1.** Sequences of the shRNA target regions and cloned constructs.

| **Vector** | **Broad Institute Clone ID** | **Target Sequence** | **Oligonucleotides, 5′-3′** |
|---|---|---|---|
| shMEN1-5 | TRCN0000040138 | CCGAGTACAGTCTGTATCAAA | FOR Accggccgagtacagtctgtatcaaagttaatattcatagctttgatacagactgtactcggtttttg |
| | | | REV Aattcaaaaaaccgagtacagtctgtatcaaagctatgaatattaactttgatacagactgtactcggc |
| shMEN1-6 | TRCN0000040139 | ATTTCGGGCACCGCATATTAA | FOR accgggctgtacctgaaaggatcatagttaatattcatagctatgatcctttcaggtacagctttttg-3′ |
| | | | REV Aattcaaaaaagctgtacctgaaaggatcatagctatgaatattaactatgatcctttcaggtacagcc |
| Non-targeting shRNA | | | FOR Accggcctaaggttaagtcgccctcggttaatattcatagccgagggcgacttaaccttaggtttttg |
| | | | REV Aattcaaaaaacctaaggttaagtcgccctcggctatgaatattaaccgagggcgacttaaccttaggc |

## Lentiviral Transduction

HEK293T cells were transfected with the lentiviral plasmid, the HIV-1 derived packaging plasmid p8.91, and the plasmid encoding the VSV-G envelope glycoproteins with help of polyethylenimine (PEI, Polysciences, Hirschberg an der Bergstraße, Germany). Viral supernatant was harvested 48 h and 72 h later. Cells were resuspended in viral supernatant and spinoculated at 2900 rpm for 2 h at 4 °C. The transfection efficiency was analyzed by FACS Canto II (BD Biosciences, Franklin Lakes, NJ, USA) 3–5 days later.

**Apoptosis Measurement**

On the day 4 after transduction the viable $RFP^+$ cells were sorted with help of the Se3 Cell Sorter (Biorad, USA). After 72 h culturing in complete medium the cell death was measured by Annexin V/PI staining by incubation with Annexin-V-FITC (BD Biosciences) and Propidium Iodide (Sigma-Aldrich, St. Lois, MO, USA). The cell death was measured with help of FACS Canto II, and analyzed with FlowJo software (BD Biosciences ). The specific apoptosis (SA) was calculated as SA,% = 100 × (AE − AC)/(100 − AC), where AE and AC are apoptosis in experimental and control groups, respectively.

**Cell Viability Analysis**

Sensitivity of the cell lines to VTP50469 (Selleckchem, Houston, TX, USA) was determined by metabolic MTT assay. Cells were seeded in triplicates into 96 well plates at a density of $2 \times 10^4$ cells per well in complete medium. Cells were treated with 2-fold serial dilutions of VTP50469 with the highest concentration of 20 μM. Solvent control wells were incubated with dimethyl sulfoxide (DMSO), at concentration corresponding to lowest drug dilution and without vehicle. Positive control wells were treated with 5 μg/mL puromycin (#540222, Merck). Cells were incubated at standard conditions for 5–6 days, followed by addition of 25 μL of the 5 mg/ml MTT solution (Thiazolyl Blue Tetrazolium Bromide, Sigma-Aldrich) for 2 hours at normal culture conditions. Then, 100 μl lysis buffer (20% SDS, 50% dimethylformamide, 2 % acetic acid, 0.15 mM HCl, pH 4.7) was added and after an overnight incubation at 37 °C, the optical densities (OD) were measured at 570 nm wavelength using the SpectraMax 250 microplate reader (Molecular Devices, San Jose, CA, USA) with help of the SoftMax Pro 3.0 software (Molecular Devices; RRID: SCR_014240). Percentage of growth inhibition at a given drug concentration was calculated as $(1 - OD_{drug} - OD_{puromycin}) / OD_{DMSO} \times 100$. The half maximal inhibitory concentration was calculated using GraphPad Prism software (RRID: SCR_002798).

**Competitive Growth Assay**

Flow cytometry for measurement of growth dynamics was performed using the FACSCanto (BD Biosciences). Percentages of $RFP^+$ cell populations were analyzed using the BD FACSDiva Software (RRID:SCR_001456) or Flow Jo Software (RRID: SCR_008520). For growth dynamics experiments, the percentage of $RFP^+$ cells was measured every 3 days. First measurement was performed 4–5 days post transduction and the percentage of $RFP^+$ cells was set as 100 % and used for normalization of results of following measurements.

**CRISPR/Cas9 Gene Editing and Analysis of the Dynamics of CRISPR-Induced InDels**

The crRNAs targeting 2nd exon of MEN1 (crRNA_MEN1_KO_1: 5′-atgatagacaggtcggccac-3′; crRNA_MEN1_KO_3: 5′-gaacgttggtagggatgacg-3′) and control crRNAs complementary to the 3rd TNNI3 intron (crRNA_TNNI3_control_1: 5′-ctatccctaagcaagtccga-3′; crRNA_TNNI3_control_2: 5′-acgtgggcccaaatctggac-3′) were selected with help of Custom Alt-R™ CRISPR-Cas9 guide RNA on-line tool (https://eu.idtdna.com/site/order/designtool/index/CRISPR_CUSTOM; 09.06.2022). The crRNAs, tracrRNA, and Alt-R® S.p. Cas9 Nuclease V3 were obtained from IDT (Leuven, Belgium). To assemble RNP complexes 200 μM of each tandem crRNA and 200 μM tracrRNA were mixed at equimolar ratio, heated at 95 °C 2 min and cooled down at room temperature. Once the mix has cooled, 450 pmol of each of the two different crRNAs:tracrRNA complexes were added to 150 pmol in total of Cas9 per nucleofec-tion. The RNP mix was incubated in the dark at room temperature for 15min. In the meantime, $5 \times 10^6$ cells was washed once in PBS and centrifuged at 1200 rpm for 5min. The cell pellet was resuspended in 100 μl of SF nucleofection buffer and transfected with help of 4D-Nucleofector® X- (Lonza, Basel, Switzerland). Typically, $5 \times 10^6$ cells were nucleofected with RNP complexes using 100 μL cuvettes. Transfection protocols are according to cell line (L363 :EH100; NALM6: CV104; RS4;11: DS120). Nucleofected cells were incubated in complete medium at normal conditions. Medium was changed every three days to maintain cell density of $0.5–1.0 \times 10^6$ per ml. Starting from the day 4 the cells were harvested with 7 day intervals. gDNA was extracted from up to $10 \times 10^6$ cells with help of DNeasy Blood & Tissue kit (Qiagen, Hilden, Germany).

To amplify edited regions, we used PCR primers targeting flanking sequences (TNNI3_fwd: 5′-tctgcccttctaaaccctc-3′; TNNI3_rev: 5′-tccaactaccgcgcttatg-3′; MEN1_NUCL_fwd: 5′-accgacaaaagaggggaag-3′; MEN1_NUCL_rev: 5′-ggtgaggttgatgatttggag-3′). The primers were synthesized by Biomeres.net GmbH (Ulm, Germany). The gDNA was amplified with help of GoTaq DNA Polymerase (Promega, Madison, WI, USA) in the presence of 5% DMSO and the amplified fragments we visualized on 1% agarose gel and purified with help of QIAquick gel extraction kit (Qiagen). The DNA fragments were sequenced by Eurofins (Eurofins Scientific SE, Luxembourg City, Luxembourg). To assess the spectrum of the InDels, the Sanger chromatograms were analyzed with help of Interference of CRISPR Edits (ICE) CRISPR Analysis tool (https://ice.synthego.com) Synthego Corporation (Redwood City, CA). This method confer analysis of the indels, generated by CRISPR-editing at the resolution level of next generation sequencing. To assess dynamics of potentially inactivating indels we used ICE parameter "Knockout Score", which represents the proportion of cells that have either a frameshift or indel exceeding 21 bp. To control potentially pro-apoptotic effect of CRISPR-induced double strand break or to exclude selection of TP53-deficient clones, which is an intrinsic set-back of CRISPR-dependent DNA editing, as control we edited 3rd intron of TNNI3.

**Statistical analysis**

All statistical analysis was carried out using PRISM9 for Mann-Whitney-Test, non-linear and simple linear regression and multiple paired t-test and Excel to normalize results and calculate the mean value of replicates.

**Immunoblotting**

Cells were lysed in SDS sample buffer (62.5 mM Tris-HCl (pH 6.8), 2 % SDS, 10 % Glycerin, 50 mM DTT, 0.01% bromphenol blue solved in water with per 10ml lysis buffer one protease inhibitor cocktail tablet (Roche Diagnostics International AG, Basel, Switzerland)) and boiled for 5–10 minutes at 100 °C. The proteins were separated by SDS-PAGE and electrophoretically transferred to a 0.45 µm nitrocellulose membrane (Amersham Pharmacia, Piscataway, NJ, USA). Membrane was blocked for 15 minutes at 37 °C in 5 % non-fat dried milk/TBS and primary antibodies (Table S2) were incubated at 4 °C overnight. Membrane was washed twice in TBS-T (0.5% Tween-20) and once in TBS (5 minutes per washing step). Horseradish peroxidase-conjugated secondary antibody was diluted 1:5000–1:10,000 (goat anti-rabbit, #31460, Thermo Fisher Scientific, Waltham, MA, USA; RRID: AB_228341) in 5 % non-fat dried milk/TBS-T or TBS-T only and membrane was incubated for 1 h at RT. Washing of the membrane was repeated as before and protein bands were visualized by addition of SuperSignal West Dura Extended Duration Substrate (Thermo Fisher). TUBB was used as loading controls.

For analysis of KMT2A (300 kDa) the sample buffer was supplemented with 6M urea. The protein separation was performed on 6% gel, followed by overnight electroblotting.

**Table S2.** List of primary antibodies.

| Primary Antibody | Species | Product Number | Dilution Factor | RRID |
|---|---|---|---|---|
| MEN1 | rabbit | #6891, Cell Signaling Technology | 1:1000 | AB_10858216 |
| TUBB | rabbit | #6046, Abcam | 1:400.000 | AB_2210370 |
| KMT2A/MLL1 | rabbit | #14689 | 1:1000 | AB_2688009 |
